# Uncovering Congenital Hypothyroidism in Adulthood: A Case Study Emphasizing the Urgency of Screening and Clinical Awareness

**DOI:** 10.7759/cureus.45611

**Published:** 2023-09-20

**Authors:** Ahmad Alam, Hamid Ashraf, Kaynat Khan, Absar Ahmed

**Affiliations:** 1 Rajiv Gandhi Centre for Diabetes and Endocrinology, Jawaharlal Nehru Medical College, Aligarh Muslim University, Aligarh, IND; 2 Department of Medicine, Jawaharlal Nehru Medical College, Aligarh Muslim University, Aligarh, IND; 3 Department of Radiodiagnosis, Jawaharlal Nehru Medical College, Aligarh Muslim University, Aligarh, IND

**Keywords:** anemia, macro-orchidism, delayed diagnosis, short stature (ss), universal newborn screening, congenital hypothyroidism

## Abstract

Congenital hypothyroidism (CH) is an endocrine disorder primarily diagnosed during the neonatal period through routine screening. Screening programs have been established in most developed countries. However, routine neonatal screening is not available in India, and the mainstay for diagnosis is clinical awareness. In this report, we present a case of CH diagnosed for the first time in a 20-year-old male who sought medical attention at our hospital’s emergency department due to fever and altered sensorium. This case report elucidates the implications of a delayed diagnosis of CH, shedding light on the pivotal role of neonatal screening and the need for enhanced awareness within the healthcare community and among families.

## Introduction

Thyroid hormone (TH) is pivotal in orchestrating various physiological processes, encompassing energy metabolism, growth, and neurodevelopment. Congenital hypothyroidism (CH) is an inborn disorder that results in low levels of TH, leading to impaired development and functioning of body tissues. The disorder is typically permanent in most affected infants, and it arises from either abnormal development of the thyroid gland or a defect in the synthesis of TH. The incidence of CH ranges from roughly 1 in 2,000 to 1 in 4,000 newborns and is one of the most common preventable causes of intellectual disability worldwide [1]. Due to its high prevalence, initial asymptomatic presentation, and risk of irreversible cognitive impairment on delayed diagnosis, routine newborn screening (NBS) is implemented in many countries worldwide, accounting for detecting most congenitally hypothyroid neonates. However, NBS is yet to be universal in some less developed countries. Without a universal screening program, delay in diagnosis and treatment is still a common problem [2,3]. Here, we report a case of CH that went undiagnosed for more than 18 years until the patient visited a tertiary care hospital for an unrelated medical condition. The case emphasizes the critical need to develop protocols for screening all babies for this potentially curable condition. Until such standards are implemented, physicians must maintain a heightened level of awareness for clinical indicators of CH to promptly diagnose and treat affected infants, thereby preventing severe neurological complications.

## Case presentation

**Figure 1:**
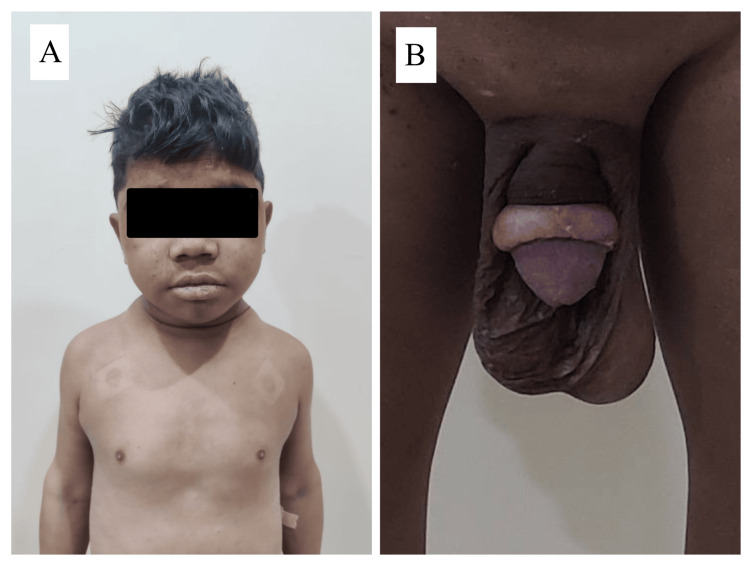
(A) Myxoedematous facies. (B) Enlarged testicular volume with absence of pubic hair.

A young adult male, aged 20, was brought to the emergency room with a five-day history of fever and altered sensorium. He had signs of meningeal irritation, and cerebrospinal fluid analysis suggested tubercular meningitis. He was prescribed anti-tubercular therapy and corticosteroids, as recommended by national guidelines. As part of the comprehensive evaluation, the medical team also noticed that he had features of hypothyroidism in the form of short stature, puffiness of the face, skin dryness, a protuberant abdomen, and delayed relaxation of the ankle reflex. The thyroid gland was not palpable. His sexual maturity rating indicated the absence of pubic hair and a bilateral testicular volume of 25 mL, suggesting macro-orchidism. Additionally, the stretched penile length was measured to be 5 cm (Figure 1). The mother informed that his scholastic performance was poor, so he discontinued his education and engaged in domestic tasks.

Investigations revealed World Health Organization grade 2 (moderate) anemia. Peripheral smear picture showed normocytic and normochromic red blood cells. The renal and liver function tests were normal. His thyroid profile suggested primary hypothyroidism, with total T3 0.1 nmol/L, total T4 2.2 nmol/L, and thyroid-stimulating hormone (TSH) >100 mIU/L (Table 1). Ultrasonography of the neck revealed bilateral small lobes of the thyroid (right 0.12 cc and left 0.2 cc). According to the Greulich-Pyle method, his bone age was six, suggesting delayed skeletal maturity.

**Table 1:** Biochemical characteristics of the patient. TIBC = total iron binding capacity; T4 = thyroxine; T3 = triiodothyronine; TSH = thyroid-stimulating hormone; TPOAb = thyroid peroxidase antibody; LH = luteinizing hormone; FSH = follicle-stimulating hormone

Parameter	Value	Reference range
Hemoglobin (g/dL)	9.4	14–18
Serum iron (µg/ dL)	70	60–150
TIBC (µg/dL)	364	250–400
Reticulocyte count (%)	0.8	0.5–2.0
T3 (nmol/L)	0.1	1.2–2.8
T4 (nmol/L)	2.2	60–160
TSH (mIU/L)	>100	0.36–5.4
TPOAb (IU/mL)	0.2	0–9
LH (IU/L)	0.4	1.2–8.6
FSH (IU/L)	1.6	1.2–19.2
Testosterone (ng/dL)	26.4	175–781
Prolactin (ng/mL)	22.8	2.6–13.1

**Figure 2:**
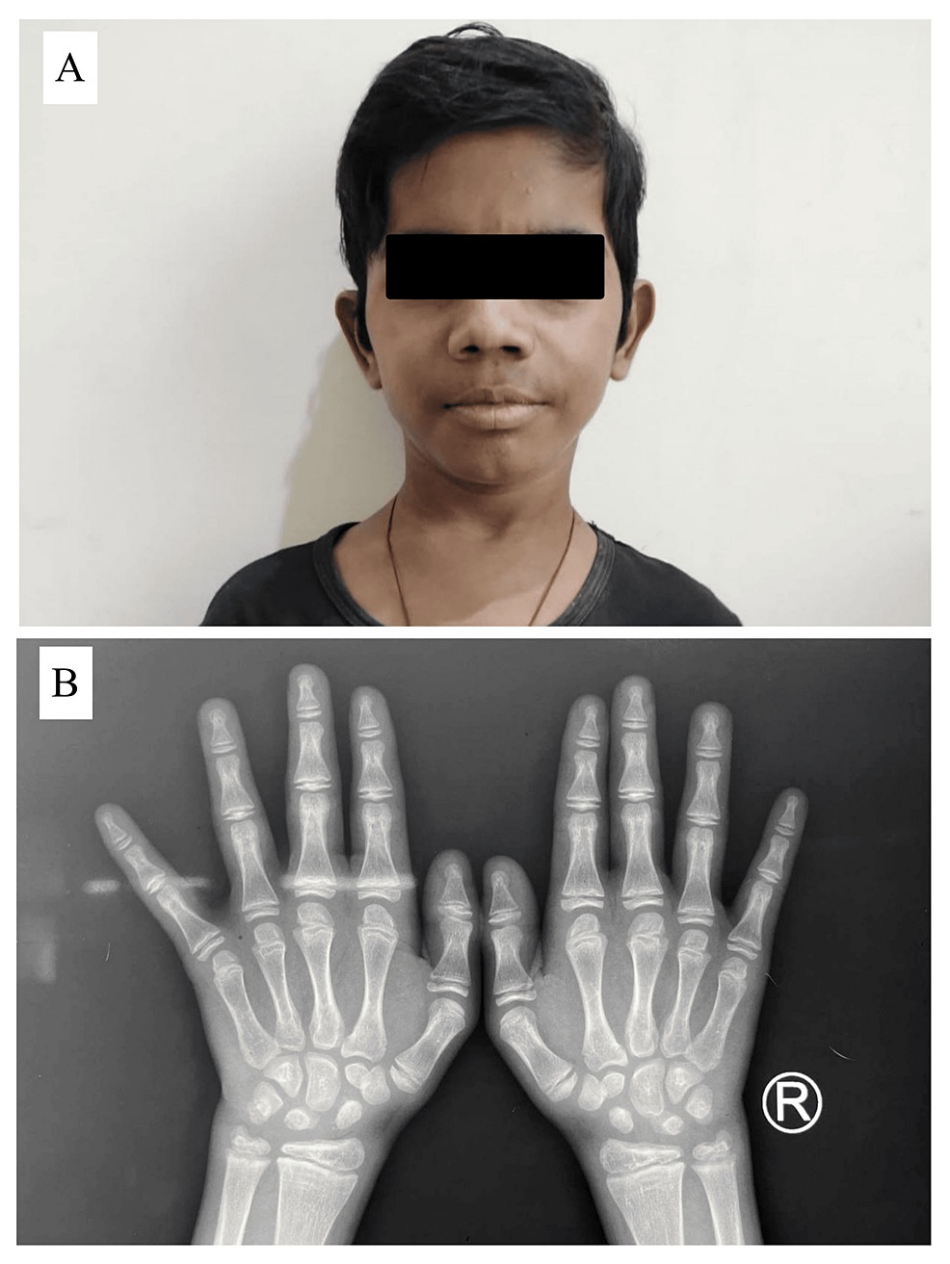
(A) Resolution of myxoedematous changes. (B) Post-treatment radiograph matches the reference bone age of an eight-year-old boy.

Given his history of long-standing untreated hypothyroidism, he was started on low-dose thyroxine at 25 µg. This dosage was escalated stepwise until the patient’s blood thyroid function results were consistently within the normal range, which was achieved at a dosage of 100 µg/day. The patient has been in follow-up for the last six months. The treatment has resulted in the resolution of myxoedematous changes and the advancement of bone age (Figure 2).

## Discussion

The clinical picture of CH varies depending on the age at which it is presented. It is common for newborns to have hyperbilirubinemia for more than three weeks. Additional symptoms include lethargy, feeding difficulty, a hoarse cry, and constipation. The most common signs on initial examination are macroglossia, dry skin, a wide posterior fontanelle, hypothermia, and an umbilical hernia. Thyroid dyshormonogenesis, characterized by a defect in TH production, can present as a palpable goiter. Short stature, gross/fine motor incoordination, decreased intelligence quotient, speech disorders, and attention deficits may occur later in life [4].

Our patient presented with myxedematous changes, anemia, short stature, and macro-orchidism. The clinical manifestations of myxedematous characteristics encompass periorbital puffiness, macroglossia, hoarseness of voice, and nonpitting edema. They are due to increased glycosaminoglycan deposition in soft tissues due to TSH excess [5].

Anemia is a common but generally underestimated clinical condition associated with hypothyroidism. TH stimulates the proliferation of erythrocyte precursors directly and indirectly by increasing erythropoietin synthesis. The most common type is normocytic anemia, with macrocytic or microcytic anemia occurring less commonly. Anemia in hypothyroidism may be caused by bone marrow depression, decreased erythropoietin production, or concomitant deficiency of micronutrients [6]. Additionally, reduced gastric acid secretion and decreased intestinal motility contribute to insufficient absorption of iron, vitamin B12, and folic acid [5].

Short stature is a common manifestation of long-standing untreated CH. Reduced growth hormone (GH) secretion and GH-mediated insulin-like growth factor 1 production are the reasons for inadequate linear growth in children with hypothyroidism. TH also directly affects the epiphyseal growth plate, stimulating chondrocyte differentiation to hypertrophic chondrocytes, which is essential for the growth of long bones [7].

Macro-orchidism in primary hypothyroidism is caused by high circulating levels of TSH, which act directly on follicle-stimulating hormone receptors, increasing the number of Sertoli cells and causing testicular enlargement. This phenomenon is referred to as specificity spillover. Because there is no activation of luteinizing hormone receptors, there is no Leydig cell hyperplasia and hence no increased testosterone production, which explains the lack of pubic hair, axillary hair, and penile enlargement [8].

Delayed diagnosis of CH is still a significant problem in less developed countries. The factors include the absence of comprehensive NBS programs, limited parental awareness, and instances of clinicians failing to diagnose the condition due to low clinical suspicion. Untreated hypothyroidism in the initial two to three years of life, a time of critical brain dependence on TH, leads to compromised neurocognitive development. Seth et al., in their study of 94 children with hypothyroidism diagnosed at the age of ≥5 years, discovered that CH was the underlying cause in over one-third of the patients [3]. The fact that our case was not identified until adulthood underscores the gravity of the underlying problem. Sukumar et al., in their study of CH, found four patients presenting late (>18 years) out of a total of 16 cases of CH diagnosed over a duration of three years, emphasizing nationwide awareness and screening [9].

The Indian Council of Medical Research National Task Force Team on NBS initiated a multi-centric study in India from 2007 to 2012. This study screened over one lakh neonates born across the country. The findings indicate a significantly elevated incidence of CH throughout India, with a rate of 1 in 722 [10]. The meta-analysis by Anne et al. also reiterates that India has a higher prevalence of CH than the global estimates [11].

## Conclusions

In an era when universal neonatal screening for CH is being implemented, it is unfortunate that CH is being diagnosed in adulthood. Our case report shows that even patients with severe hypothyroidism with a classic clinical picture can go years without being diagnosed until they develop life-threatening symptoms that force them to seek medical attention. This underscores the need for increased consciousness among the general public and primary healthcare providers in nations that have yet to adopt universal NBS.

## References

[1] Ford G, LaFranchi SH (2014). Screening for congenital hypothyroidism: a worldwide view of strategies. Best Pract Res Clin Endocrinol Metab.

[2] Ihsan I, Rini EA (2017). Delayed diagnosis of congenital hypothyroidism in an adolescent results in avoidable complications: a case report. Paediatr Indonesiana.

[3] Seth A, Aggarwal V, Maheshwari A (2012). Hypothyroidism in children beyond 5 y of age: delayed diagnosis of congenital hypothyroidism. Indian J Pediatr.

[4] Rastogi MV, LaFranchi SH (2010). Congenital hypothyroidism. Orphanet J Rare Dis.

[5] Bhansali A, Gogate Y (2015). Hypothyroidism. Clinical Rounds in Endocrinology.

[6] Szczepanek-Parulska E, Hernik A, Ruchała M (2017). Anemia in thyroid diseases. Pol Arch Intern Med.

[7] Bhansali A, Aggarwal A, Parthan G, Gogate Y (2016). Disorders of growth and development: clinical perspectives. Clinical Rounds in Endocrinology.

[8] Philip R, Saran S, Gutch M, Gupta K (2013). An unusual case of precocious puberty and macroorchidism. Thyroid Res Pract.

[9] Sukumar SP, Balachandran K, Jayakumar Jayakumar, Kamalanathan S, Sahoo JP, Das AK, Halanaik D (2013). Congenital hypothyroidism - an usual suspect at an unusual age: a case series. Indian J Endocrinol Metab.

[10] (2018). Newborn screening for congenital hypothyroidism and congenital adrenal hyperplasia. Indian J Pediatr.

[11] Anne RP, Rahiman EA (2022). Congenital hypothyroidism in India: a systematic review and meta-analysis of prevalence, screen positivity rates, and etiology. Lancet Reg Health Southeast Asia.

